# Supplementary material for: Research on assessment method of maximum distributed generation hosting capacity in distribution system with high shares of renewables and power electronics
Source: PLoS One. 2024 Nov 4;19(11):e0313164. doi: 10.1371/journal.pone.0313164 (PMC11534231; doi:10.1371/journal.pone.0313164)
Supplement: Attachment — Submitted filename: Response to Reviewers.pdf [file pone.0313164.s002.pdf]

Dear Editors and Reviewers,

We greatly appreciate your professional suggestions and the time and effort you dedicated to providing feedback on our manuscript. Based on your valuable input, we have extensively revised our manuscript. Modifications have been highlighted in yellow in the "revised manuscript with track changes". The detailed point-by-point responses are listed below:

1、It is necessary to read the full text carefully and check carefully to avoid low-level grammatical errors and improper words and phrases. For example, there is a problem with incomplete display of image labels in line 393.

Thanks for your suggestion. We have check the full text carefully and tried our best to polish the language in the revised manuscript. These changes will not influence the content and framework of the paper, so we did not list the changes. In addition, the incomplete display of image labels has been corrected. (page 14, line 423)

2、In this paper, the linear simplified evaluation model of maximum carrying capacity is developed as a point of contribution, what is the difference between him and other assessment models in the literature, please add the explanation.

Thank you for your suggestion. We would like to provide a detailed explanation of the issues concerning the linear simplification evaluation model.

The maximum DG hosting capacity assessment method proposed in this paper consists of two parts: firstly, the linear optimization model that can quickly calculate the preliminary maximum DG hosting capacity. This model is based on optimal power flow, which is the same as other assessment models in the literature; secondly, the correction of preliminary assessment results based on recursive method, which allows us to obtain more accurate evaluation results. So the contribution of our paper is to propose a integrated assessment method that combines linear optimization method and recursive method as shown in Figure 1. We apologize for not expressing it clearly.

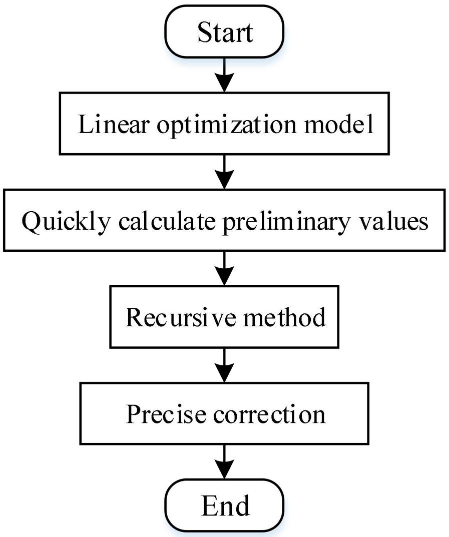

Fig 1. The integrated assessment method

The main modifications are as follows (page 2, line 57-66). Other modifications have been highlighted in yellow in the "revised manuscript with track changes".

The contributions of this paper are summarized as follows:

- An integrated assessment method for maximum DG hosting capacity is proposed that combines the linear optimization method and the recursive method. It can realize fast and accurate calculation of maximum DG hosting capacity in the distribution network.
- An improved comprehensive sensitivity index is proposed, which can accurately find the most suitable DG connection node for power correction during the recursive process.
- A safety constraint verification method based on safety region is proposed, which can realize rapid safety verification calculations and improve the calculation efficiency of recursive algorithm.

3、In the subsection "Improved comprehensive sensitivity index", the rationale for the improved comprehensive sensitivity index is not clear enough, and it is recommended that further additional clarification be provided.

Thank you for your suggestion. We have supplemented the rationale for the improved comprehensive sensitivity index. The modifications are as follows. (page

8-9, line 232-248)

In order to find the most suitable DG connection node for power correction during the recursive process, this paper proposes an improved comprehensive sensitivity index. The rationale of the improved comprehensive sensitivity is as follows.

The improved node voltage sensitivity is obtained from Eq (26).

$$\delta'_{i,p} = \frac{\delta_{i,p}}{V_{\lim} - V_{i,t}} \tag{35}$$

where $V_{\lim}$ represents the voltage limit, and $V_{i,t}$ is the voltage at node i at time t.

The improved line power sensitivity is obtained from Eq (34).

$$\gamma'_{h,p} = \frac{\gamma_{h,p}}{S_{\lim} - S_{h,t}} \tag{36}$$

where $S_{\lim}$ represents the line power limit, $S_{h,t}$ is the power of line h at time t.

The improved comprehensive sensitivity of DG connection node p is as follows:

$$\xi_p = \max\left(\delta'_{i_1,p},\ldots,\delta'_{i_n,p},\gamma'_{h_1,p},\ldots,\gamma'_{h_k,p}\right), p \in N_{DG} \tag{37}$$

Under the optimization objective represented in Eq (20), when the recursive process requires an increase in DG output power, choose the DG connection node with the smallest ξ as the power correction node; when the recursive process requires a decrease in DG output power, choose the DG connection node with the largest ξ as the power correction node. In addition, it can be seen from Eq (37) that the value of ξ_p is obtained from the sensitivity of a certain state variable. This suggests that as the DG output power increases at node p, this state variable will reach the safety boundary first. Therefore, the improved sensitivity index proposed in this paper can assist in identifying weak points in the distribution network.

4、How can the validity of the DG maximum load carrying capacity simulation results be determined, and is the methodology used in this paper different from the results obtained by other literature methods?

(1)The validity of the simulation results for the maximum DG hosting capacity is determined by the following two aspects:

A. Safety verification of simulation results

Adjust the DG output power according to the simulation results presented in this paper. The state variables of the distribution network are as follows:

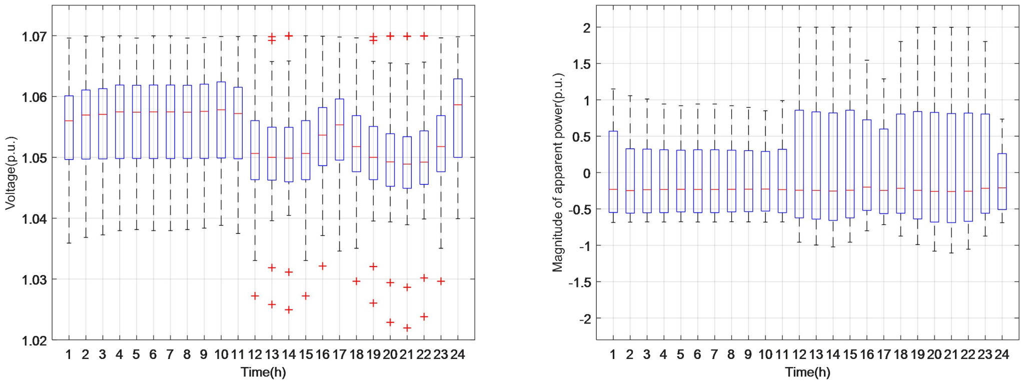

Fig 2. Distribution network state variables in scenario 1

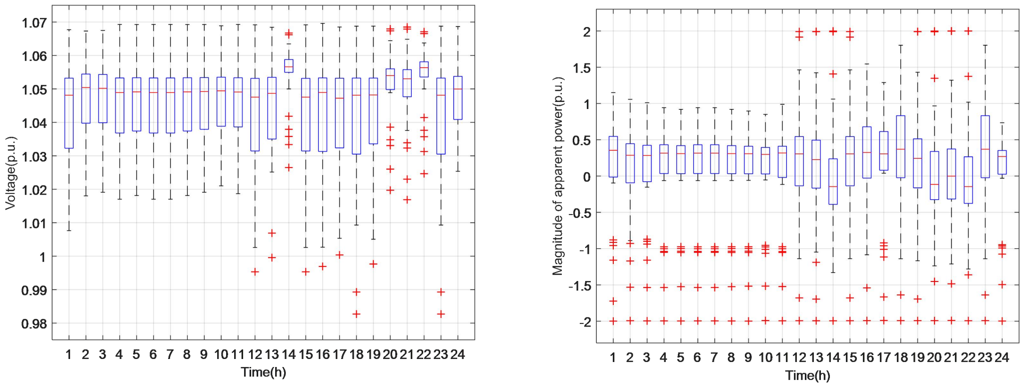

Fig 3. Distribution network state variables in scenario 2

It can be observed from Fig 2 and Fig 3 that the distribution network state variables are all within the safety constraints, proving the safety of the simulation results.

B. Accuracy verification of the simulation results

Comparison is made between the integrated assessment method and the linear optimization method commonly used in other literature. The DG output power is adjusted according to the simulation results of the two methods, respectively, and the state variables of the distribution network are calculated. D_V and D_S are then used to evaluate the distance between the operating state and the safety boundary of the distribution network. The expressions for D_V and D_S are as follows:

$$D_{S,t} = \frac{\sum_{h=1}^{k} \left| S_{\lim} - S_{h,t} \right|}{k} \tag{40}$$

where k is the number of lines in the distribution network, $S_{\lim}$ represents the line power limit, and $S_{h,t}$ is the power of line h at time t.

$$D_{V,t} = \frac{\sum_{i=1}^{n} \left| V_{\lim} - V_{i,t} \right|}{n} \tag{41}$$

where n is the number of nodes in the distribution network, $V_{\lim}$ represents the voltage limit, and $V_{i,t}$ is the voltage at node i at time t.

From the above equation, it can be seen that the smaller the values of D_V and D_S, the closer the operating state of the distribution network is to the safety boundary, indicating higher accuracy in the assessment results. The D_V and D_S of the two methods are shown in the figure below:

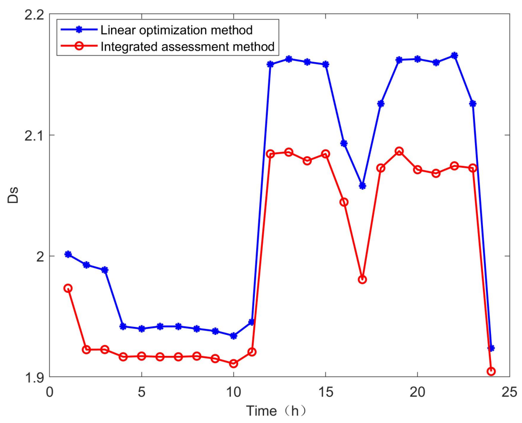

Fig 4. The D_S in scenario 1

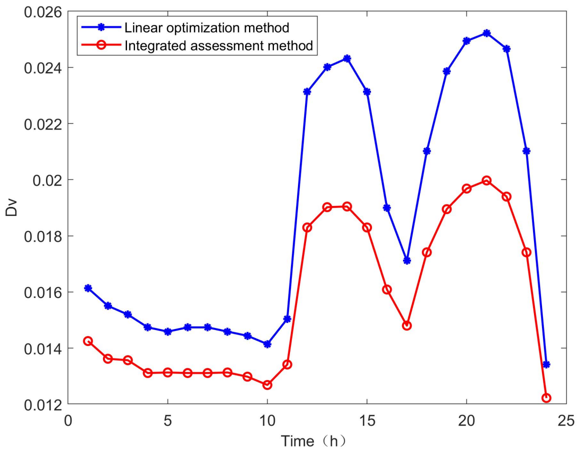

Fig 5. The D_V in scenario 1

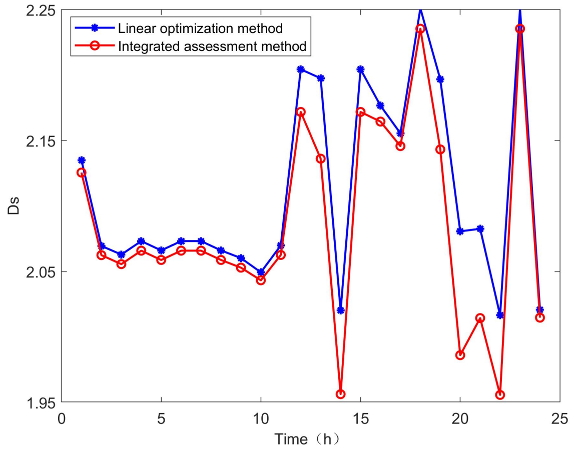

Fig 6. The D_S in scenario 2

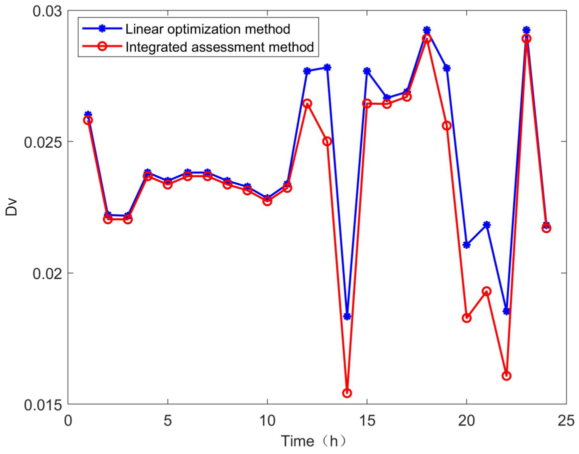

Fig 7. The D_V in scenario 2

It can be observed from Fig 4 to Fig 7 that the simulation results of the proposed method in this paper have lower values of DV and DS, indicating higher accuracy of

the assessment method proposed in this paper. This also validates the effectiveness of the DG maximum load hosting capacity simulation results.

(2)The difference between the methodology used in this paper and the results obtained by other literature method

The comparison of the assessment results between the method proposed in this paper and other literature methods is shown in Fig 8.

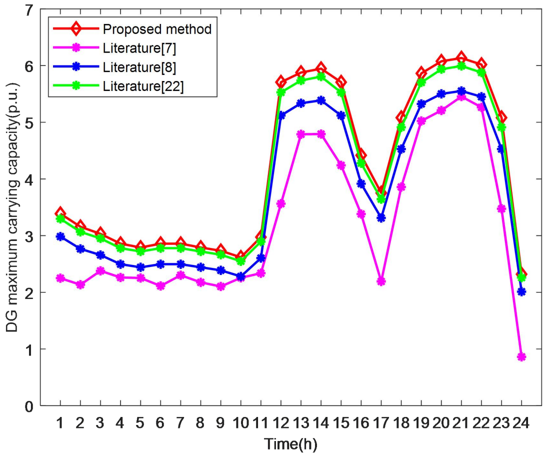

Fig 8. The comparison of the assessment results between the method proposed in this paper and other literature methods

The literature [7] adopts an iterative calculation method with increasing customer penetration, conducting DG hosting analysis by gradually raising the customer penetration rate. However, this method did not consider the operational characteristics of each node. The literature [8] applied Monte Carlo simulation method to evaluate the maximum DG hosting capacity of distribution networks, but the accuracy of the calculation results is affected by the upper limit of customer penetration. The literature [22] adopts a linear optimization method. The following two situations will occur when using linear optimization method to calculate the maximum DG hosting capacity.

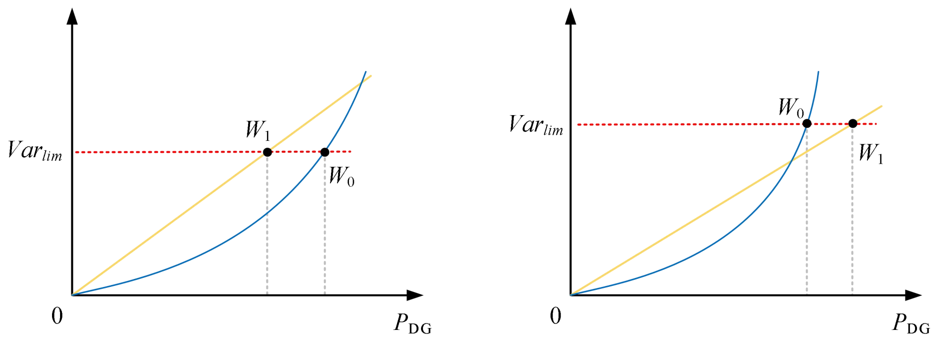

(a)Situation 1 (b)Situation 2

Fig 9. The integrated assessment method

Var_{lim} represents the limit of the distribution network state variables, W_0 represents the true value of the maximum DG hosting capacity, and W_1 represents the calculation result of linear optimization method.

It can be seen from Fig 8 and Fig 9 that the calculation results of other literature methods may be either conservative or aggressive. The method proposed in this paper can reduce the conservatism of the calculation results by adjusting the magnitude of the DG correction power ΔP_{DG} in the recursive process. The safety constraint verification during the recursive process ensures that the calculation results do not become too aggressive and violate safety constraints.

5、In the subsection "Comparison of different sensitivity indexes", the traditional sensitivity indexes are briefly described to give the reader a basic understanding.

Thank you for your suggestion. We have supplemented the introduction of the traditional sensitivity index in the subsection "Comparison of different sensitivity indexes". The revisions are as follows. (page 13-14, line 406-413)

This subsection compares the performance of different sensitivity indexes in searching for the most suitable DG connection node for power correction. The process of searching for the power correction node using traditional sensitivity indices is as follows:

Step 1: Find the state variable var_i with the smallest difference from the limit.

Step 2: Calculate the sensitivity of var_i with respect to the injected power at each

DG connection node according to Eq (26) and Eq (34).

Step 3: According to the traditional sensitivity index in step 2, determine the DG connection node for power correction.

Thank you again for your positive comments and valuable suggestions to improve the quality of our manuscript.

Yours Sincerely,

Corresponding author: Jingyu He

Apr 13 2024

E-mail: hjy397766@gmail.com
