# Supplementary material for: Research on assessment method of maximum distributed generation hosting capacity in distribution system with high shares of renewables and power electronics
Source: PLoS One. 2024 Nov 4;19(11):e0313164. doi: 10.1371/journal.pone.0313164 (PMC11534231; doi:10.1371/journal.pone.0313164)
Supplement: Attachment — Submitted filename: Response to Reviewers.docx [file pone.0313164.s003.docx]

Dear Editors and Reviewers,

Thank you for your valuable comments and suggestions on our manuscript. We appreciate the time and effort you have spent in reviewing our work. Based on your valuable input, we have extensively revised our manuscript. Modifications have been highlighted in yellow in the "revised manuscript with track changes". The detailed point-by-point responses are listed below:

**Reply to Reviewer #2**

Comment 1:

Do the 'safety constraints' and 'safety region' in Fig. 3 refer to the same thing? If they are two different things, this needs to be made clear.

Response 1:

Thank for your suggestion. According to the definition in Eq(2), the upper bound *a_i,max_* and the lower bound *a_i,min_* of the distribution network state variable for both the 'safety constraints' and 'safety region' are derived from Eq(7) and Eq(8). However, the *g_i_*(***W_t_***) for the 'safety constraints' is obtained through power flow calculation, while the *g_i_*(***W_t_***) for the 'safety region' is calculated using the safety domain model. (page 9, line 280-282)

Comment 2:

Adding a few sentences on the organization of the paper would be helpful.

Response 2:

Thank you for your suggestion. We have added a description of the organization of the paper. (page 2-3, line 66-71)

The remainder of this paper is organized as follows: Section 2 introduces the concepts of the distribution network operating point and the maximum DG hosting operating point. Section 3 presents the model for assessing maximum DG hosting capacity. Section 4 presents the proposed methodology, describing a two-step method that combines the linearization method and the recursive method. Section 5 presents the test system, numerical results, and discussion.

Comment 3:

It would be better if the manuscript could provide direction on future research or the next steps following this study's completion in the conclusion. Please consider providing some guidance in this area.

Response 3:

Thank you for your suggestion. We have added an introduction about the direction on future research. (page 14, line 440-442)

To accurately assess the maximum DG hosting capacity in the distribution network, this paper proposes an two-step method that combines the linearization method and the recursive method. Firstly, the maximum DG hosting capacity in the distribution network is preliminarily calculated using linear optimization method, and then the preliminary calculation results are precisely corrected using recursive method. During the recursive process, an improved comprehensive sensitivity index is proposed to find the most suitable DG connection node for power correction. The improved comprehensive sensitivity index can accurately reflect the impact of different DG connection nodes on the distribution network state variables and achieve precise identification of weak points. Additionally, this paper proposes a safety constraint verification method based on security regions, which enhances the calculation speed of the recursive algorithm. Finally, the proposed method was tested and validated on the IEEE 33-bus distribution network. The simulation results indicate that the proposed method can accurately assess the maximum DG hosting capacity in the distribution network. The improved comprehensive sensitivity exhibits significant performance improvement over traditional sensitivity index in enhancing the accuracy of calculating the maximum DG hosting capacity. To further enhance the robustness of the assessment results, the next step of this study will focus on researching an evaluation method for DG hosting capacity in distribution networks that considers various uncertainties.

**Reply to Reviewer #3**

Comment 1:

Some discussion of the right-hand side of Eq(2) would help the reader understand the equation.

Response 1:

Thank you for your suggestion. We have added an introduction to the right-hand side of Eq (2). The revisions are as follows: (page 3, line 89-91)

where **Ω_S_** is the distribution network safety region, *g_i_*(***W_t_***) represents the value of the distribution network state variable *a_i_* when the operating point is ***W_t_***, *a_i,max_* and *a_i,min_* are the upper and lower limits of the distribution network state variable *a_i_*, respectively.

Comment 2:

Please consider citing references to simplify the derivation process of Eq(17)–Eq(19).

Response 2:

Thank you for your suggestion. We have simplified the derivation process of Eq (17) - Eq.(19) as per your recommendation. (page 6, line 178-179)

Comment 3:

The reviewer notices that the parameters *V_lim_* and *S_lim_* are used multiple times throughout the paper. In which scenarios do these parameters represent upper bounds, and in which scenarios do they represent lower bounds?

Response 3:

Thank you for your suggestion. The theoretical analysis in the this paper indicates that an increase in DG output power will lead to a rise in node voltage and an increase in reverse power on the feeder. Therefore, for the assessment of DG hosting capacity, *V_lim_* should be set as the upper limit of voltage, and *S_lim_* should be set as the upper limit of reverse power on the line. When the assessment target is the hosting capacity for distribution network loads or electric vehicle integration, *V_lim_* should be set as the lower limit of voltage, while *S_lim_* should be set as the upper limit of forward power on the line.

**Changes to the reference list**

In the reference list, the format of references [4] and [12] has been modified. Reference [23] was a paper from Hunan Electric Power, but it could not be found on Google Scholar, so it has been replaced with relevant current references.

Thank you again for your positive comments and valuable suggestions to improve the quality of our manuscript.

Yours Sincerely,

Corresponding author: Jingyu He

Oct 9 2024

E-mail: hjy397766@gmail.com
